# Predictors of Poor Sleep Quality in Elderly Individuals in Western Iran: A Population-Based Cross-Sectional Study

**DOI:** 10.34172/jrhs.2025.177

**Published:** 2024-12-25

**Authors:** Zahra Cheraghi, Nasrin Shirmohammadi, Razieah Ilukhani, Mojtaba Tayebi, Parvin Cheraghi, Mohadeseh Sadri

**Affiliations:** ^1^Department of Epidemiology, School of Public Health, Hamadan University of Medical Sciences, Hamadan, Iran; ^2^Modeling of Noncommunicable Diseases Research Center, Hamadan University of Medical Sciences, Hamadan, Iran; ^3^Department of Biostatistics, Hamadan University of Medical Sciences, Hamadan, Iran; ^4^Health Deputy, Hamadan University of Medical Sciences, Hamadan, Iran; ^5^Behavioral Disorders and Substance Abuse Research Center, Hamadan University of Medical Sciences, Hamadan, Iran; ^6^Department of Public Health, School of Public Health, Hamadan University of Medical Sciences, Hamadan, Iran; ^7^Iranian Research Center on Aging, University of Social Welfare & Rehabilitation Sciences, Tehran, Iran

**Keywords:** Sleep-wake disorders, Iran, Elderly, Cross-sectional studies

## Abstract

**Background:** Poor sleep quality in the elderly is a prevalent issue that can significantly impact overall health and quality of life. This study aimed to assess the prevalence of sleep disorders and the factors contributing to poor sleep quality among older adults in Western Iran.

**Study Design:** This is a cross-sectional study.

**Methods:** This study involved 403 elderly people. The following tools were employed to collect data: the Pittsburgh Sleep Quality Index (PSQI), the Leisure and Pleasure Activities Database (a quality-of-life tool), the standardized Depression, Anxiety and Stress Scale (DASS-21), and the Abbreviated Mental Test (AMT)for cognitive assessment. A backward stepwise selection method was employed to finalize the variables for multiple logistic regression analysis.

**Results:** The overall prevalence of poor sleep quality was 44.7%. With each one-point increase in stress, the likelihood of experiencing poor sleep quality increases significantly (adjusted OR: 1.09, *P*<0.001). The number of children in the household was found to have a protective effect against poor sleep quality (adjusted OR=0.63, *P*=0.008). Furthermore, elderly individuals working as housekeepers had higher odds of poor sleep quality than those employed elsewhere (adjusted OR=7.45, *P*=0.005).

**Conclusion:** A significant association was observed between elevated stress levels and poor sleep quality. Interestingly, the presence of children in the household appeared to offer a protective effect. Conversely, individuals in household management roles faced a dramatically increased risk of poor sleep quality. These findings offer preliminary evidence for the potential effectiveness of early interventions and prevention strategies designed to improve sleep quality and reduce social frailty in the elderly.

## Background

 Sleep is widely recognized as one of the fundamental pillars of circadian rhythms and plays a crucial role in various bodily functions, including growth, learning, and memory enhancement.^1^ Sleep disorders refer to disturbances in sleep patterns or changes in sleep-related behavior. Individuals who suffer from sleep disorders may have difficulties falling asleep, waking up too early, or having interrupted sleep, often leading to dissatisfaction with sleep quality.^2^ Research suggests that sleep disorders and poor sleep quality can negatively impact both physical and mental health.^3^

 Insomnia is a common problem, affecting a significant percentage of the population at some point in their lives. It is estimated that around 30%-45% of people worldwide suffer from insomnia, with prevalence increasing with age. Studies indicate that around 57% of older adults experience sleep problems.^4^ According to a 2014 report by the National Sleep Foundation, approximately 35% of African-American adults rated their sleep quality as poor.^5^ Furthermore, a study conducted on African-American seniors found that nearly 13% sleep less than four hours per night, and 14.5% wake up three or more times during the night, with women tending to wake up more frequently than men.^6^

 Sleep problems in older adults are often mistakenly attributed to aging, when in fact, they are typically caused by factors such as underlying medical conditions, medications, depression, anxiety, and mobility limitations, rather than changes in circadian rhythms.^7,8^ Common sleep disorders in older people include insomnia, circadian rhythm disorders, excessive daytime sleepiness, sleep aponia, and restless legs syndrome.^8^ Poor sleep is associated with higher rates of illness, increased hospitalization, and reduced quality of life.^9^ Insufficient sleep significantly increases the risk of falls, as well as conditions such as depression and dementia. While aging leads to some changes in sleep quality and structure, numerous sleep problems are due to external factors, including health and social support.^10^ Sleep disorders impose a significant burden on patients and healthcare systems, underscoring the need for more effective and accessible healthcare solutions. However, few national studies have been conducted in this area, and there is currently no compelling evidence regarding the underlying causes of sleep disorders in the elderly.

 This study aimed to assess the prevalence of sleep disorders and their contributing factors among older adults in Hamadan Province during 2023-2024, highlighting the pressing public health concern of sleep health in the elderly population.

## Methods

 The cross-sectional study comprised 403 elderly individuals (aged 60 and above) residing in Hamedan, Iran, conducted during the latter half of 2023. The researchers used a stratified cluster sampling method, with health clusters serving as clusters. These clusters were classified by socio-economic status into poor, medium, and good categories. Urban and rural centers were randomly selected from these groups, with sample sizes allocated according to the elderly population in each center. Trained health workers and caregivers conducted face-to-face interviews using a structured questionnaire that took about 30 minutes to complete.

 The required sample size was calculated based on the results of a study conducted in Iran, which reported a 54.1%prevalence of poor sleep quality.^11^ With a maximum achievable error (precision level) was 0.12, and after adjusting for cluster sampling (design effect: 1.78), the final sample size was determined to be 403 participants, considering the design effect. Inclusion criteria were local residents aged 60 years and older, not taking medication that could affect cognitive function, and willing to participate.

 The data collection process consisted of five sections: demographic information, the Pittsburgh Sleep Quality Index (PSQI),^12^ a quality of life tool (Leipad),^13^ a standardized Depression, Anxiety, and Stress Scale (DASS-21),^14^ and a cognitive assessment (Abbreviated Mental Test, AMT).^15^ Demographic data such as gender, age, marital status, education level, and number of children were self-reported.

 The PSQI assesses sleep quality in older individuals over the previous month. It evaluates seven dimensions: overall sleep quality, sleep latency, actual sleep time, sleep efficiency, sleep disturbances (such as night waking), use of sleep medications, and daytime functioning. This scale consists of 19 items, with each dimension scored from 0 (no problem) to 3 (very serious problem). The individual scores are summed to yield a total score ranging from 0 to 21, with higher scores indicating poorer sleep quality. A score of 6 or higher indicates inadequate sleep. The validity and reliability of this questionnaire for elderly individuals have already been established in previous studies.^16^

 The DASS-21, developed by Lovibond et al, was used to assess depression, anxiety, and stress levels. The questionnaire consists of 21 items: 8 related to depression, 7 to anxiety, and 6 to stress. Respondents indicate their feelings over the past week based on four categories, and response options of ‘very bad’, ‘bad’, ‘good’, and ‘very good’. Participants completed the questionnaire in an interview, with researchers clarifying any unclear wording. The validity of this questionnaire has been demonstrated in previous studies.^17,18^

 Finally, the AMT was used to detect cognitive impairment in older people. This brief cognitive assessment is less dependent on the participant’s level of education compared to tests like the Mini-Mental State Examination (MMSE). The Persian version of the AMT has been validated in Iran and has shown adequate discriminant validity in distinguishing between individuals with and without cognitive impairment, with a sensitivity of 92.15%, specificity of 81.5%, and Cronbach’s alpha of 0.76.^19^ The chi-squared test was used to compare proportions between subgroups at the 5% significance level. Multiple logistic regression models were used to assess the impact of independent factors on the outcome (poor sleep quality), using the backward stepwise selection method for final variable selection. In this approach, the significance and removal levels were set at 0.1 and 0.2, respectively. All analyses were performed using Stata 17 statistical software.

## Results

 The results indicated that 63.5% of participants lived in urban areas, with a higher proportion of women (56.8% women vs. 43.2% men). Most participants were married (74.9%), and the average age was 68.93 (6.8) years. A significant number had low levels of education, with 51.4% being illiterate and 31.5% having only primary education. Health assessments revealed that 64.3% of participants had chronic illnesses, although 94.0% reported a good quality of life. Mental health assessments revealed that 53.6% have normal levels of depression, 30.5% have moderate depression, and 15.4% have severe depression. In terms of anxiety, 49.9% were normal, with 24.1% reporting severe anxiety. Stress levels showed that 62.0% have normal stress, 22.6% have moderate stress, and 14.6% have severe stress (See Table 1).

**Table 1:** Demographic Characteristics of the Subjects

**Variables**	**Number**	**Percent**
Habitat		
Rural	147	36.5
Urban	256	63.5
Gender		
Male	174	43.2
Female	229	56.8
Material status		
Single	6	1.5
Have a wife	302	74.9
Divorced/Widow	95	23.6
Education		
Illiterate	207	51.4
Elementary	127	31.5
Middle-school	26	6.4
High-school	19	4.7
Academic	19	4.7
Unknown	5	1.2
History of chronic disease		
Yes	259	64.3
No	144	35.7
Depression		
Normal	216	53.6
Moderate	123	30.5
Severe	62	15.4
Unknown	2	0.5
Anxiety		
Normal	201	49.9
Moderate	97	24.1
Severe	103	25.6
Unknown	2	0.5
Stress		
Normal	250	62.0
Moderate	91	22.6
Severe	59	14.6
Unknown	3	0.8

 The overall prevalence of poor sleep quality (classified as “very bad” category) was 44.7%. Among the dimensions of sleep quality, poor sleep duration had the highest prevalence at 29%, followed by difficulty falling asleep (sleep latency) at 6.5%, and the necessity of using sleeping medications at 6.5%, respectively (See Table 2).

**Table 2:** Distribution of sleep quality dimensions

**Sleep quality dimension**	**Very Good**		**Relatively Good**		**Relatively Bad**		**Very Bad**	
	**Number**	**Percent**	**Number**	**Percent**	**Number**	**Percent**	**Number**	**Percent**
Subjective sleep quality	80	19.9	276	68.5	40	9.9	7	1.7
Sleep latency	115	28.5	183	45.4	79	19.6	26	6.5
Sleep duration	286	71.0		0.0	0	0.0	117	29.0
Habitual sleep efficiency	311	77.2	62	15.4	23	5.7	7	1.7
Sleep disturbances	44	10.9	270	67.0	82	20.3	7	1.7
Use of sleeping medication	285	70.7	64	15.9	28	6.9	26	6.5
Daytime dysfunction	236	54.0	121	58.6	5	1.2	41	10.2

 The analysis of poor sleep quality by different factors reveals significant findings. Among participants, poor sleep quality was more common among urban residents (46.9%) than in rural residents (40.8%), though this difference was not statistically significant (*P* = 0.239). A higher percentage of women (49.3%) reported poor sleep quality compared to men (37.9%), and this difference was statistically significant (*P* = 0.023). The presence of children also appeared to significantly affect sleep quality: 95.0% of those with 1-2 children reported poor sleep quality, while those with three or more children (83.1%) and those with no children (45.5%) reported better sleep quality. However, the *P*-value (0.712) did not indicate any significant differences between these groups.

 In addition, 90.0% of older individuals with only a primary school education reported poor sleep quality (*P* = 0.027). Although a history of chronic disease did not significantly affect sleep quality (*P* = 0.488), a striking 90.9% of those reporting a low to moderate quality of life had poor sleep (*P* < 0.001). Furthermore, poor sleep quality was particularly higher and statistically significant *(P* < 0.001) among those with moderate to severe levels of depression (54.6%), anxiety (62.5%), and stress (58.6%), as depicted in Table 3.

**Table 3:** Poor sleep quality by related factors

**Variables**	**Number**	**Percent**	***P*** ** value**
Area habitant			0.239
Rural	60	40.8	
Urban	120	46.9	
Gender			0.023
Male	66	37.9	
Female	112	49.3	
Living status			0.213
Living alone	31	51.7	
Just with spouse	69	41.3	
With both spouse and children	55	42.0	
Just with children	25	55.6	
Children			0.712
No children	5	45.5	
1-2	34	95.0	
≥ 3	135	83.1	
Education			0.027
Elementary	154	90.0	
Middle school/high school	17	9.9	
Academic	7	36.8	
Chronic disease			0.488
Yes	119	45.9	
No	61	42.4	
Quality of life			0.001
Low/Moderate	20	90.9	
High	149	43.2	
Depression			0.001
Normal	94	45.4	
Abnormal	113	54.6	
Anxiety			0.001
Normal	62	34.8	
Abnormal	116	62.5	
Stress			0.001
Normal	82	41.4	
Abnormal	116	58.6	

 The analysis presented in Table 4 outlines the impact of several factors on sleep quality. Stress emerged as a significant contributor, with an adjusted odds ratio (OR) of 1.09 (95% CI: 1.04-1.14), indicating that for each one-point increase in stress, the likelihood of experiencing poor sleep quality significantly rises (*P* < 0.001). In terms of habitat, individuals living in urban areas had lower odds of poor sleep quality compared to those in rural areas, with an OR of 0.54 (95% CI: 0.25-1.18), although this finding did not reach statistical significance (*P* = 0.122*)*. Quality of life was negatively correlated with poor sleep quality, yielding an OR of 0.97 (95% CI: 0.93-1.00), but this association was not statistically significant (*P* = 0.085). Interestingly, the number of children in a household was found to have a protective effect against poor sleep quality, with an OR of 0.63 (95% CI: 0.45-0.89), which was statistically significant (*P* = 0.008). Lastly, being a housekeeper rather than actively employed was linked to a significantly higher risk of poor sleep quality, with an OR of 7.45 (95% CI: 1.81-30.55), with a *P *value of 0.005.

**Table 4:** The role of some factors on the poor sleep quality

**Variables**	**Adjusted OR (95% CI)**^a^	***P*** ** value**
Stress (per one score)	1.08 (1.02, 1.44)	0.008
Habitat (urban vs. rural)	0.48 (0.22, 1.05)	0.067
Number of children (per one)	0.37 (0.18, 0.78)	0.009
Housekeeper vs. employed	8.32 (2.04, 33.95)	0.003
Anxiety (per one score)	1.04 (0.98, 1.11)	0.130

*Note.* OR: Odds ratio; CI: Confidence interval. ^a^ Backward stepwise selection was used for variable selection in the multiple logistic regression.

## Discussion

 The findings of this study indicate the significant prevalence of sleep disorders among the elderly population. Stress, household size (particularly the number of children), and the role of the housekeeper were identified as the most salient factors associated with this disorder. Notably, all three of these factors are considered amenable to modification.

 Sleep disorders in the elderly are a widely recognized issue with a variety of underlying causes, including hormonal changes, chronic illnesses, medication use, and psychological conditions associated with aging. As individuals age, natural changes in sleep patterns may result in various sleep-related problems, including insomnia, daytime sleepiness, and decreased sleep quality. The present study, which aimed to assess sleep quality in elderly Iranians and identify its predictive variables, found that nearly half of the elderly participants report poor sleep quality. In a review study, Jalali et al reported a prevalence of sleep disorders among Iranian seniors of 49%.^20^ Similarly, Gulia and Kumar’s study indicated that 20%-60% of individuals aged 60 and above worldwide suffer from sleep disorders.^21^ It is estimated that over half of older adults are affected by sleep disorders, which significantly impact their quality of life.^22^

 In the present study, the dimensions of poor sleep quality with the highest prevalence were insufficient sleep duration, disruption of daily functioning, delayed sleep onset, and the necessity of using sleeping pills. A substantial body of research supports the finding that elderly individuals typically experience a reduction in the number of hours slept compared to younger age groups. Furthermore, older adults frequently encounter disturbances during their sleep, which not only affects their quality of life but also impacts their overall health and daily functioning. The consequences of poor sleep quality in older adults extend beyond the individual to the social and economic spheres. Poor sleep can lead to cognitive disorders, daytime drowsiness, frequent naps, decreased daytime performance, reduced functional independence, increased dependency, reliance on sedative medications and their misuse, as well as heightened risks such as accidents and falls. Additionally, it can pose various socio-economic consequences for elderly individuals, their families, caregivers, and the wider community.^23-26^

 The results of the current study on the predictors of poor sleep quality in the elderly indicated that female gender, loneliness, stress, depression, low education level, and low quality of life are significantly correlated with reported poor sleep quality among participants. Anxiety also emerged as a significant factor influencing sleep quality among older adults. The daily experience of worry and negative thinking can elevate sympathetic nervous system activity, which in turn can lead to disturbances in sleep. Numerous studies have indicated that older adults with elevated levels of anxiety tend to experience more severe sleep disturbances compared to their counterparts.^25-27^

 It has been well-demonstrated that depression can not only disrupt daily life but also directly affect sleep, thereby making the onset of sleep disorders a common symptom of depression in the elderly. A considerable proportion of older adults with depressive disorders also experience insomnia or excessive daytime sleepiness.^26,28,29^ Moreover, psychological and social stressors such as financial pressures, loneliness, and the loss of loved ones, can have a profound impact on the sleep of the elderly.^30,31^ Effective management of stress and the enhancement of coping skills have been demonstrated to significantly improve sleep quality.^32^

 Furthermore, gender is a key factor in the prevalence of sleep disorders among older adults. The available evidence suggests that elderly women are more likely to experience sleep difficulties than men. This discrepancy may be attributed to hormonal fluctuations, particularly during menopause, as well as elevated rates of anxiety and depression among women. Furthermore, women are more likely to experience feelings of loneliness and emotional distress, which can have a detrimental impact on their sleep quality.^33,34^

 It is noteworthy that the quality of life is another important factor when examining sleep disorders. Sufficient and high-quality sleep contributes to an enhanced quality of life and positively impacts physical and mental health.^35^ The available research indicates that enhancing sleep quality among older adults can positively affect their quality of life and overall health. Implementing strategies such as regulating sleep patterns, avoiding caffeinated beverages in the evening, and creating a conducive sleep environment can help improve sleep quality. Furthermore, these factors can contribute to a decline in the quality of life.^32^

 One of the key strengths of the present study was its examination of sleep quality in elderly individuals across a diverse range of provinces, including both urban and rural elders from a variety of social and cultural backgrounds. This diversity allowed the research team to investigate the role of residence on sleep quality. However, this study also had several limitations, including its cross-sectional design, which inherently restricts the ability to assess causal relationships. It is recommended that future research utilize longitudinal studies. Additionally, the current study only included elderly individuals residing in the community as it was conducted in urban and rural areas. Consequently, information regarding elderly individuals living in long-term care facilities was not included in the study. It would be beneficial to conduct further studies to ascertain whether sleep interventions can delay the onset of cognitive decline in these individuals.

**Highlights**

- Poor overall sleep quality was reported by 44.7% of the elderly.
- Poor sleep duration was most prevalent dimension, affecting 29% of participants.
- Each stress point increase boosts poor sleep risk by 9%.
- Having children in the household has a protective effect against poor sleep quality.
- Housekeepers face a 7.45 times higher risk of poor sleep quality.

## Conclusion

 This study showed a significant association between elevated stress levels and poor sleep quality in elderly individuals. Interestingly, the presence of children in a household appeared to offer a protective effect, whereas individuals in household management roles faced a dramatically increased risk of poor sleep quality. The findings offer preliminary evidence for the effectiveness of early intervention and prevention strategies aimed at improving sleep quality and reducing social frailty among older adults.

## Acknowledgments

 We would like to thank all the elderly individuals from Hamadan province who participated in this study.

## Competing Interests

 The authors declare no competing interests.

## Ethical Approval

 This study was approved by the Ethical Committee of the Vice-Chancellor of Research and Technology, Hamadan University of Medical Sciences (IR.UMSHA.REC.1402.614, Code 140209147950). The funders had no role in the study design, data collection, and analysis, decision to publish, or manuscript preparation.

## Funding

 Not applicable.
